# Assessment of neopterin and indoleamine 2,3‐dioxygenase activity in patients with seasonal influenza: A pilot study

**DOI:** 10.1111/irv.12677

**Published:** 2019-09-06

**Authors:** Alex Pizzini, Katharina Kurz, Janine Santifaller, Christoph Tschurtschenthaler, Igor Theurl, Dietmar Fuchs, Günter Weiss, Rosa Bellmann‐Weiler

**Affiliations:** ^1^ Department of Internal Medicine II, Infectious Diseases, Pneumology, Rheumatology Innsbruck Medical University Innsbruck Austria; ^2^ Division of Biological Chemistry Biocenter Innsbruck Medical University Innsbruck Austria

**Keywords:** biomarker, IDO, indoleamine 2,3‐dioxygenase, Influenza, Neopterin, outcome

## Abstract

**Background:**

Seasonal influenza is an important cause of morbidity and mortality worldwide. Immune activation after stimulation with interferon‐gamma leads to increased production of neopterin but also results in increased tryptophan catabolism through indoleamine 2,3‐dioxygenase (IDO). Our pilot study determined neopterin serum levels and IDO activity in patients with influenza infection and investigated whether neopterin is linked to clinical outcome parameters (mortality ≤30 days, acute cardiac events (ACE) length of hospitalization, ICU admission).

**Methods:**

Neopterin concentrations were analyzed in serum samples of 40 patients with a confirmed diagnosis of influenza infection and in‐hospital treatment for >24 hours. Data were compared to values of 100 healthy blood donors and 48 age‐matched pneumonia patients. In a subgroup of 14 patients, tryptophan and kynurenine concentrations, as well as kynurenine‐to‐tryptophan ratio, were analyzed.

**Results:**

In all influenza patients, neopterin concentrations were increased and significantly higher compared to those determined in patients with pneumonia and healthy controls. Positive correlations between the duration of hospitalization and neopterin were found. Significantly higher levels of kynurenine, kynurenine‐to‐tryptophan ratio, and lower levels of tryptophan were seen in influenza patients compared to healthy controls.

**Conclusions:**

Neopterin seems to be related to the course of the disease and could be a valuable biomarker to identify patients at an elevated risk of a worsened outcome; however, further prospective validation studies are needed to support the here presented preliminary results.

## 1 INTRODUCTION

Seasonal influenza is an important cause of morbidity and mortality worldwide, with an annual incidence rate estimated between 5% and 10% in adults and 20% and 30% in children, resulting in about 290 000‐650 000 deaths per year.^1^ Especially infants, older adults and patients with chronic illnesses like cardiovascular disease or immunosuppression are at risk.^2^, ^3^ Continuous variations of circulating seasonal influenza virus subtypes, dual or repeated infections during one season and limited effectivity of vaccination are major challenges.^4^, ^5^, ^6^

The pteridine neopterin is a marker of cell‐mediated immune activation, which is biochemically inert and whose half‐life in the human organism is mainly affected by renal excretion.^7^, ^8^, ^9^ Activation of monocytes/macrophages after stimulation with interferon‐gamma (IFN‐γ) leads to increased production of neopterin.^8^, ^9^ However, activation of the immune system via IFN‐γ also results in increased tryptophan catabolism through the kynurenine pathway. Degradation of tryptophan to kynurenine is catalyzed by indoleamine 2,3‐dioxygenase (IDO).^10^ Indoleamine 2,3‐dioxygenase is inducible by pro‐inflammatory cytokines, in particular IFN‐γ.^11^, ^12^, ^13^

Increased neopterin levels are observed during viral infections, and previous studies have shown that serum neopterin levels combined with C‐reactive protein (CRP) can differentiate between viral and bacterial etiologies in acute respiratory tract infections.^14^, ^15^, ^16^ Increased levels of neopterin have previously also been associated with adverse outcomes in different diseases including viral infections.^8^, ^16^, ^17^, ^18^ Similarly, tryptophan metabolism is able to slow down cellular immune response, for example, during infections, and thus, the determination of tryptophan metabolites may hold promise to predict outcomes in viral infections.^19^

Since influenza antigen bed‐side tests have limited sensitivity and polymerase chain reaction (PCR)‐based methods are expensive and often not available, cheaper and more reliable biomarkers for diagnosis, and risk stratification of patients with influenza infection are desired.^20^

The aim of this pilot study was to investigate neopterin levels and IDO activity in sera of hospitalized patients with influenza infection and to analyze correlations with clinical outcome parameters in a small real‐life cohort. We also questioned if discrimination between influenza and community‐acquired pneumonia (CAP) is possible by assessing neopterin serum levels.

## 2 PATIENTS AND METHODS

### 2.1 Subjects

We retrospectively analyzed eligible subjects by reviewing the electronic medical records of patients, who were treated at the Department of Internal Medicine II, at the Medical University Innsbruck between December 2012 and February 2017. Patients were classified as having influenza infection if either the antigen test or the PCR test was positive.

Patients between 18 and 95 years were included if influenza‐assay (antigen test or real‐time PCR) results were positive and neopterin serum levels were routinely assessed. In‐hospital treatment of >24 hours was necessary.

**Figure 1:**
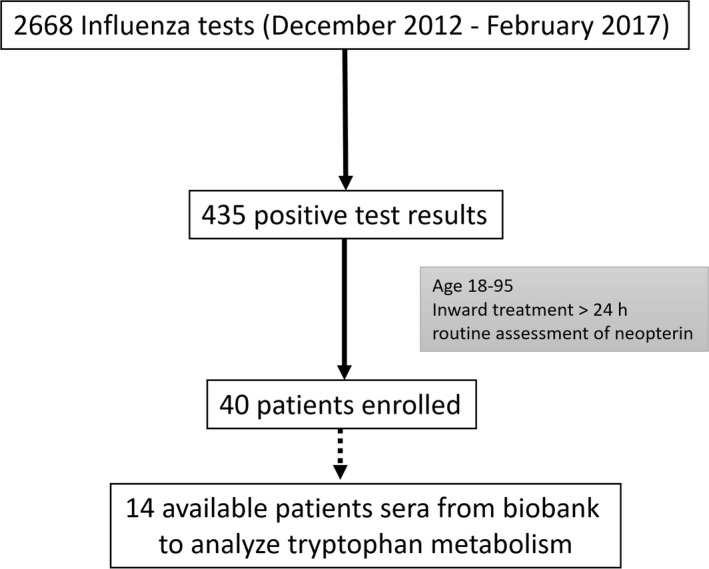
Algorithm for the inclusion of patients

Forty patients (15 female, 25 male) met our inclusion criteria and were enrolled in the analysis. The detailed demographic description is shown in Table 1. The algorithm upon the selection of patients is presented in Figure 1. In all patients, the following data were analyzed at admission to the hospital: standard laboratory test including complete blood count, renal and hepatic function tests, CRP, neopterin, and high‐sensitive troponin‐T (hsTroponinT). We also recorded the duration of hospitalization, admission to the intensive care unit (ICU), and 30‐day mortality. In patients presenting with a hsTroponinT level above the upper limit of the normal (ULN > 14 ng/L; n = 14), acute cardiac events (within 30 days) defined as myocardial ischemia, arrhythmogenic events, and heart failure requiring therapy were recorded.

**Table 1:** Demographics of the cohort – patients with influenza, CAP, and healthy controls

Parameter		Influenza (n = 40)	CAP (n = 48)	Healthy controls	*P*‐Value
No. of patients		40	48	100	
Age (y)		60.4 ± 21.6	62.75 ± 18.92	49 ± 11.4	
Gender male^b^		25 (62.5)	30 (62.5)	58 (58)	
Virus type					
Influenza A^b^		30 (75)			
Influenza B^b^		10 (25)			
Laboratory parameters	Reference				
Neopterin (nmol/L)	0‐10	49.46 ± 24.98	45.6 ± 58.73	5.94 ± 1.58	**<.01**
CRP (mg/dL)^a^	0‐0.5	3.91 ± 4.11	13.75 ± 9.22	/	**<.01**
WBC (1000/L)^a^	4‐10	7.7 ± 3.66	10.35 ± 4.46	/	**<.01**
Creatinine (mg/dL)^a^	0.51‐0.95	0.98 ± 0.32	1.16 ± 0.86	/	.58
hsTroponinT (ng/L)^a^	0‐14	21.54 ± 29.42	/	/	
Days of hospitalization^a^		6.78 ± 4.01			

Note

Quantitative parameters are represented as mean ± standard deviation.

Abbreviations: CAP, community‐acquired pneumonia; CRP, C‐reactive protein; hsTroponinT, high‐sensitive troponin‐T; WBC, white blood cells.

a Represents non‐normally distributed data.

b Categorical parameters are represented as total n and percentage. Statistical test applied: Mann‐Whitney *U* test for non‐normally distributed data, *t* test for normally distributed data, chi‐square test for analysis of association between categorical variables.

Bold values denote statistical significance at *P* < .05.

During in‐hospital treatment, patients were asked to voluntarily donate blood for further biochemical analyses. Of the 40 included patients, 14 agreed (35%) and tryptophan and kynurenine concentrations, as well as the kynurenine‐to‐tryptophan ratio (Kyn/Trp), were determined. Tryptophan and kynurenine concentrations were measured by high‐performance liquid chromatography (HPLC) on reversed phase, and the ratio of kynurenine to tryptophan (Kyn/Trp) was calculated to estimate IDO activity.^21^ Neopterin concentrations were determined by ELISA (BRAHMS Diagnostica).

These results were compared to a historical cohort of 100 healthy volunteers.^22^ The healthy volunteers were recruited from the Central Institute of Blood Transfusion and Immunology of the University Clinics Innsbruck after exclusion of infection or active diseases. The mean age of the cohort was 49 ± 11.4 years, and 42 participants were women. (The demographics of the cohort are described in Table 1 and were published previously 22).

To evaluate the discriminative potential of neopterin, we studied neopterin levels in an age‐matched historical cohort of 48 patients with CAP.^23^ Community‐acquired pneumonia patients were included if CAP‐related symptoms, typical respiratory auscultation sounds, and a chest x‐ray or CT scan confirming a pneumonic infiltrate were present. The cohort consisted of 18 female and 30 male patients, the mean age was 62.75 ± 18.92 years, and all patients needed in‐hospital therapy. The exact demographics of the cohort are described elsewhere.^23^

### 2.2 Statistical analyses

Mean comparison of normally distributed numeric data was performed using Student's *t* test. If Gaussian distribution was not given, the Mann‐Whitney *U* test and Kruskal‐Wallis test were applied. Baseline characteristics in terms of categorical variables were compared using chi‐square, and Fisher's exact test, where appropriate. Spearman rank correlation technique was used for analysis of monotonic associations in non‐normally distributed data. If Gaussian distribution based on Shapiro‐Wilk test and a linear relationship were given, Pearson correlation coefficient was calculated to assess the degree of correlation. The diagnostic potentials of neopterin, CRP, and WBC to differentiate between CAP and influenza were evaluated by receiver operating characteristic (ROC) analysis and its respective areas under the curve (AUC). All tests were two‐sided, and a *P*‐value of .05 indicated statistical significance. Statistical analyses were performed with the SPSS 24.0 statistical package (IBM Corp.).

### 2.3 Ethical approval

All samples and data were fully anonymized. Patients provided written informed consent prior blood storage in the biobank and consented to use their medical records and samples for research purposes. All procedures performed in the present study involving human participants were in accordance with the ethical standards of the Institutional and/or National Research Committee and with the 1964 Helsinki declaration and its later amendments and were performed after approval of the Ethics Committee of the Medical University of Innsbruck (AN2017‐0054 371/4.10).

## 3 RESULTS

### 3.1 Clinical cases

**Figure 2:**
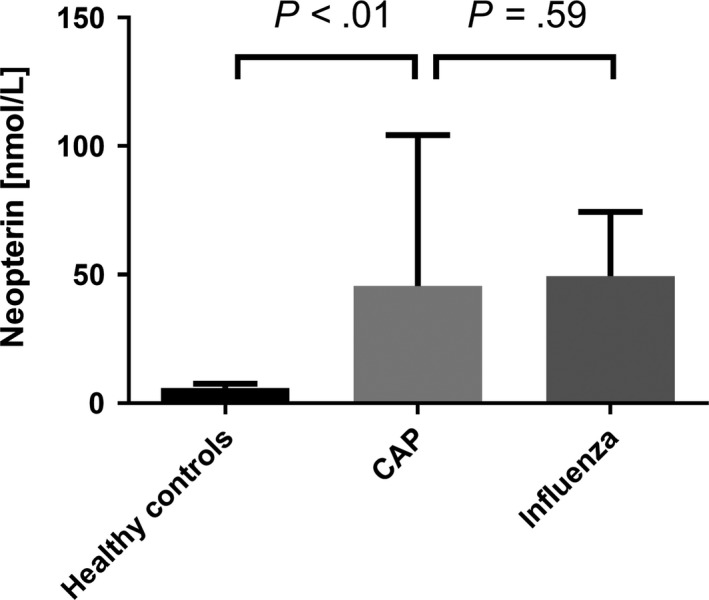
Neopterin serum concentrations in healthy controls, CAP, and influenza patients. Data are shown as mean ± standard deviation. Mean comparison of the three groups was performed by Kruskal‐Wallis test. CAP, community‐acquired pneumonia

Forty patients were included in this retrospective analysis, and the ratio of influenza A and B was 3:1. The mean age of the patients was 60.4 ± 21.6 years, and male patients accounted for 62.5% of cases (Table 1). Sixteen patients were diagnosed using a mucosal antigen test, and 24 patients were tested positive for influenza using nucleic acid amplification test (PCR). In 20 cases, the antigen test revealed false‐negative results and the diagnosis was established by PCR subsequently. Our cohort showed an elevation of CRP (mean ± standard deviation (SD): 3.91 ± 4.11 mg/dL; reference range 0‐0.5 mg/dL) and neopterin levels (49.46 ± 24.98 nmol/L; reference range 0‐10 nmol/L), while creatinine, hsTroponinT, and WBC were within the defined normal ranges (Table 1, Figure 2). When splitting the cohort into patients with either influenza virus type A or B, no significant differences between laboratory parameters were seen (Table 2).

**Table 2:** Diagnostic test methods, laboratory and prognostic parameters divided by influenza virus subtype and respective comparison between the two groups

Parameters	Influenza A (n = 30)	Influenza B (n = 10)	*P*‐Value
Diagnostic test (positive samples)			
PCR (n)	19	5	
Antigen test (n)	11	5	
AG negative/ PCR positive^b^	15 (50%)	5 (50%)	
Laboratory parameters			
Neopterin (nmol/L)	48.56 ± 22.76	52.19 ± 31.98	.70
CRP (mg/dL)^a^	3.78 ± 4.57	4.31 ± 2.40	.13
WBC (1000/L)^a^	7.92 ± 3.55	7.04 ± 4.10	.25
Creatinine (mg/dL)^a^	0.96 ± 0.25	1.06 ± 0.49	.59
hsTroponinT (ng/L)^a^	22.15 ± 31.92	19.74 ± 21.51	.96
Prognostic parameters			
Days of hospitalization^a^	6.3 ± 3.1	8.2 ± 6.0	.23
30‐day mortality^b^	0	1 (10)	
ICU^b^	1 (3.5)	1 (10)	
ACE in patients with hsTroponinT > ULN^b^	3 (27.3)	2 (66.7)	

Note

Quantitative parameters are represented as mean ± standard deviation.

Abbreviations: PCR, polymerase chain reaction (number of positive samples); AG, antigen test (number of positive samples); CRP, C‐reactive protein; WBC, white blood cells; ICU, intensive care unit; ACE, acute cardiac events; ULN, upper limit of the norm.

a Represents non‐normally distributed data.

b Categorical parameters are represented as total n and percentage. Statistical test applied: Mann‐Whitney *U* test for non‐normally distributed data, *t* test for normally distributed data.

Neopterin levels significantly correlated with creatinine (*r* = .34, *P* = .03) and hsTroponinT (*r* = .47, *P* < .01), while no significant correlations were found with CRP and WBC. The mean length of hospitalization was 6.78 ± 4.01 days. Positive correlations with the duration of hospitalization were found for hsTroponinT (*r* = .45, *P* < .01) and neopterin (*r* = .37, *P* = .02) levels at admission.

One patient suffering from influenza B died within 30 days, and two patients needed ICU treatment (influenza A:B = 1:1). Fourteen patients presented with increased hsTroponinT concentrations, and five patients had an acute cardiac event (ACE). At admission, patients with ACE showed significantly higher serum levels of neopterin compared to those with no ACE (79.16 ± 20.31 nmol/L vs 47.47 ± 17.83 nmol/L, *P* = .01), while hsTroponinT only showed a trend toward higher levels in patients with ACE (71.50 ± 54.72 ng/L vs 33.24 ± 15.63 ng/L, *P* = .08).

No significant differences in terms of laboratory parameters (CRP *P* = .1, neopterin *P* = .2, WBC *P* = .6, hsTroponinT *P* = .2) and duration of hospitalization (*P* = .9) were found between patients with positive and false‐negative influenza antigen test.

### 3.2 Tryptophan metabolism

Fourteen serum samples of patients with confirmed influenza infection (Flu A: Flu B = 13:1) were identified in our biobank and subsequently analyzed and compared to a healthy historical cohort. The exact details of the control cohort are described elsewhere.^22^

**Figure 3:**
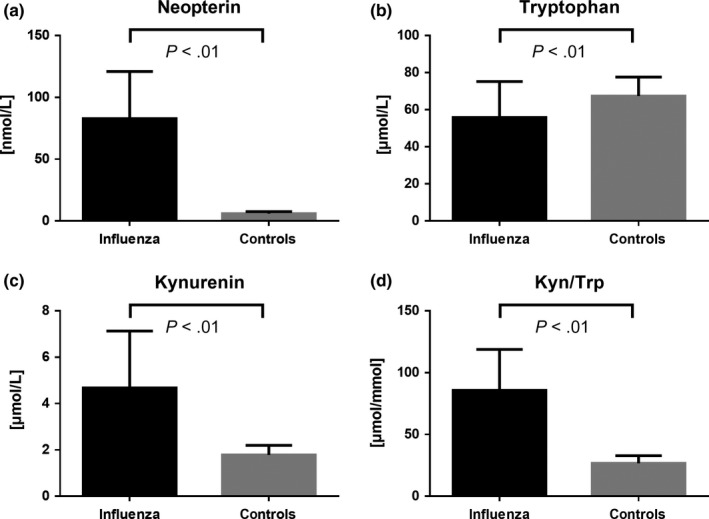
Comparison of neopterin (A), tryptophan (B), kynurenine (C), and Kyn/Trp (D) levels between influenza patients and healthy controls. Data are shown as mean ± standard deviation. Mean comparison of the two groups was performed by Mann‐Whitney *U* test. Black bars indicate influenza patients; gray bars indicate healthy controls. Kyn/Trp, kynurenine‐to‐tryptophan ratio

The mean concentrations of neopterin, tryptophan, kynurenine, and Kyn/Trp, as well as the demographic details of the sub‐cohort, are shown in Table 3. Overall, we found significantly higher levels of neopterin (*P* < .01), kynurenine (*P* < .01), Kyn/Trp (*P* < .01), and lower levels of tryptophan (*P* < .01) when comparing influenza patients to healthy controls (Figures 2 and 3).

**Table 3:** Demographics, neopterin, and tryptophan metabolism parameters in influenza‐infected patients vs. healthy controls

Parameter	Influenza (n = 14)	Controls (n = 100)	*P*
Influenza A:B	13:1		
Age (y)	63.14 ± 20.32	49 ± 11.36	**<.01**
Male^b^	5 (35.7)	58 (58)	.15
Neopterin (nmol/L)^a^	87.44 ± 36.91	5.94 ± 1.57	**<.01**
Tryptophan (umol/L)^a^	53.71 ± 20.08	67.39 ± 10.20	**<.01**
Kynurenine (umol/L)^a^	4.89 ± 2.74	1.78 ± 0.42	**<.01**
Kyn/Trp (umoL/mmoL)^a^	91.01 ± 34.36	26.66 ± 6.19	**<.01**

Note

Quantitative parameters are represented as mean ± standard deviation.

a Represents non‐normally distributed data.

b Categorical parameters are represented as total n and percentage. Statistical test applied: Mann‐Whitney *U* test for non‐normally distributed data, *t* test for normally distributed data, Kyn/Trp = kynurenine‐to‐tryptophan ratio.

Bold values denote statistical significance at *P* < .05.

Positive correlations were found between neopterin and kynurenine (*r* = .53, *P* < .01) as well as between neopterin and Kyn/Trp (*r* = .63, *P* < .01), while neopterin and tryptophan levels correlated inversely (*r* = −.25, *P* < .01).

The mean duration of hospitalization in this sub‐population of 14 influenza patients was 7.15 ± 3.96 days. One patient presented with an ACE, none of the patients needed ICU treatment or died within 30 days. No significant correlations were found between the duration of hospitalization and tryptophan (*P* = .41), kynurenine (*P* = .73) or Kyn/Trp (*P* = .83).

### 3.3 The discriminative potential of neopterin in the setting of influenza and CAP

**Figure 4:**
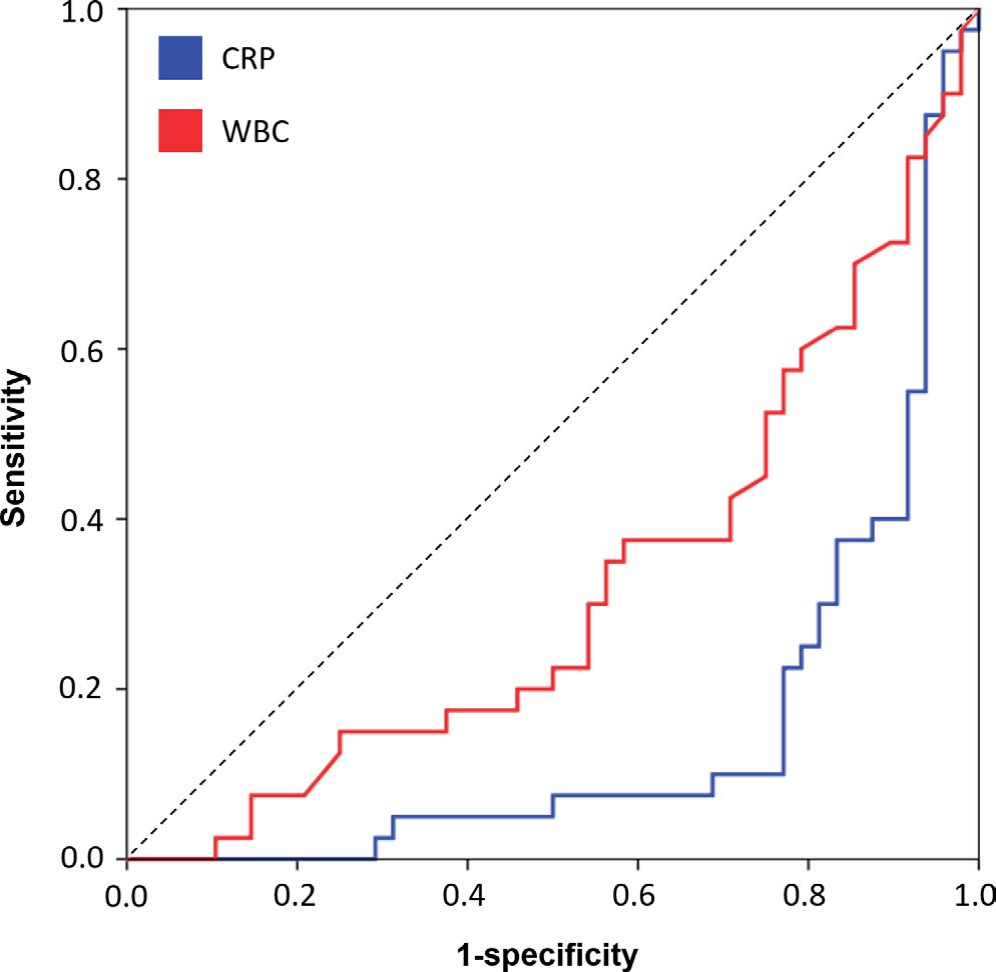
Receiver operating characteristic analysis to differentiate between CAP and influenza using the parameters neopterin, CRP, and WBC. CRP, C‐reactive protein; WBC, white blood cells

To analyze the discriminative potential of neopterin, a cohort of 48 patients with CAP was compared to the influenza patients.^23^ The two cohorts were well matched in terms of age, gender, and creatinine levels (Table 1). Community‐acquired pneumonia and WBC concentrations were significantly higher in CAP patients compared to influenza patients (*P* < .01), whereas neopterin concentrations were significantly lower (*P* < .01, Table 1, Figure 2). Receiver operating characteristic analysis for differentiation between CAP and influenza patients showed the highest AUC for CRP (AUC = 0.849), followed by WBC (AUC = 0.680) and neopterin (AUC = 0.663, Figure 4).

## 4 DISCUSSION

Influenza is an important cause of morbidity and mortality worldwide. Several inflammatory markers, which correlate with disease severity, have intensively been studied in the past.

In our pilot study, we were able to show an elevation of neopterin serum levels at the diagnosis of influenza infection. Moreover, neopterin correlated with prognostic variables, namely the duration of hospitalization, hsTroponinT levels at admission, and acute cardiac events. Also, neopterin levels can discriminate between influenza infection, healthy controls, and CAP; however, CRP and WBC revealed higher AUC.

To date, only very few studies analyzed neopterin in human influenza virus infection. Chang et al described neopterin concentrations in Chinese patients with acute dengue fever infection and compared it to other virus infections like influenza.^16^ They showed an elevation of neopterin levels above the upper limit of the norm in all viral infections. However, they found significant differences between the various etiologies with up to threefold higher concentrations in dengue patients when compared to influenza patients. They also showed the potential of neopterin as a prognostic marker in dengue fever infection, as initial levels correlated well with the duration of fever. In comparison to Chang et al, our study cohort revealed higher mean neopterin levels, which might be the result of an earlier assessment and diagnosis of the infection by us.

In a small cohort of 20 influenza patients, Kajiya et al measured neopterin levels, showing an elevation of neopterin > ULN in 80% of the patients at symptom onset (within 48 hours).^24^ During recovery (between days 5 and 14), a fast decrease of neopterin was observed (neopterin > ULN in only 1/20 patients), and normal neopterin levels were reported in all patients after day 14. The exact characteristics of the sub‐cohort are not described, and information about patient's recovery or complications during the course of the diseases are not reported either, therefore not allowing a direct comparison with our study. However, the similarities regarding neopterin elevation further support the value of this biomarker in early influenza virus assessment.

For the first time, we were able to demonstrate a simultaneous increase of neopterin as well as tryptophan catabolism (determined by the elevation of kynurenine and Kyn/Trp) during human influenza virus infection. Tryptophan catabolism is very likely due to the induction of IDO by pro‐inflammatory cytokines, in particular IFN‐γ.^11^, ^12^, ^13^ Most data about IDO induction and influenza virus infection are derived from mouse models: Yoshida et al showed a 120‐fold increase in IDO activity in murine lungs after infection with PR8 influenza virus.^25^ Huang et al investigated IDO1 knockout mice after influenza PR8 infection and showed that genetic IDO ablation led to much faster recovery after virus clearance.^26^

Pett et al analyzed the prognostic value of IDO activity in 96 hospitalized adult patients infected with the pandemic influenza A H1N1.^27^ The case‐control designed study compared patients with disease progression defined as death within 60 days, need for ICU therapy, and mechanical ventilation to patients with stable disease. High Kyn/Trp ratios were associated with a poor outcome. It is difficult to compare our data with results of this study, as inflammatory biomarkers like CRP, WBC, or neopterin were not reported and influenza A H1N1pdm09 overall was characterized by high mortality rates.^27^

There is growing evidence that elevated cell‐mediated immune response as measured by neopterin and IDO is associated with elevated cardiovascular mortality, as shown in large epidemiological studies.^17^, ^28^, ^29^ Cardiac injuries like myocarditis, myocardial infarction, and congestive heart failure are dangerous complications of influenza infection, and these events are either driven by a tropism of the virus for the heart or exacerbation of coronary artery diseases as a consequence of influenza driven inflammatory response.^2^, ^3^, ^30^ Our data revealed an association between cardiovascular complications, hsTroponinT, and neopterin serum levels in the setting of influenza virus infection, further suggesting neopterin as a potential marker to contribute to risk prediction in patients with cardiovascular disease. Obviously, large prospective validation studies are needed to draw robust conclusions in this regard.

Influenza diagnostics needed confirmation via PCR in half of the here investigated cases. Rapid influenza tests using direct antigen detection cards usually have high specificity (>98%), but moderate to low sensitivity, ranging between 29.6% and 77.8%, depending on the used commercial test.^20^, ^31^ We did not observe any differences in neopterin levels between patients with positive and negative antigen test. However, we speculate that in patients with a high clinical pre‐test probability elevated neopterin serum levels might be helpful to diagnose influenza virus infection even in the case of a false‐negative influenza antigen test. In this regard, the comparison of influenza and CAP patients revealed only a weak discriminative potential of neopterin, in contrast to CRP.

Although our preliminary results are promising, we also have to acknowledge some limitations. We determined laboratory parameters retrospectively in a relatively small cohort of severely ill patients needing hospital treatment. The exact delay between symptom onset and influenza diagnosis was not recorded, and we only used a single blood sample at admission. Longitudinal follow‐up measurements of neopterin and tryptophan metabolism were not available to assess the kinetics during the course of the disease. In addition, the cohorts, especially the healthy controls, were not perfectly age‐matched. A slight age‐related increase of neopterin and degradation of tryptophan was previously described 32; hence, larger and closer‐matched cohorts are needed for further prospective investigations. Finally, the CAP cohort was not tested for influenza; however, only patients with CAP‐related symptoms, typical respiratory auscultation sounds, and a chest X‐ray or CT scan confirming a pneumonic infiltrate were included, therefore lowering the probability of influenza patients wrongly being classified as CAP patients.

## 5 CONCLUSION

In summary, we were able to show an elevation of neopterin serum levels as well as a simultaneous induction of IDO as represented by an elevated Kyn/Trp in all patients with influenza virus infection. Neopterin seems to be related to outcome variables, while in our small cohorts, the performance to differentiate between CAP and influenza was poor. Neopterin might be a valuable biomarker to identify patients at an elevated risk of a worsened outcome; however, further prospective validations studies are needed to support the here presented results.

## Data Availability

All relevant data are within the paper. The data used to support the findings of this study are available from the corresponding author upon request (rosa.bellmann-weiler@i-med.ac.at). Data cannot be shared publicly because of privacy concerns.
